# Activation modes in biocatalytic radical cyclization reactions

**DOI:** 10.1093/jimb/kuab021

**Published:** 2021-03-01

**Authors:** Yuxuan Ye, Haigen Fu, Todd K Hyster

**Affiliations:** Department of Chemistry, Princeton University, Princeton, NJ 08544, USA; Department of Chemistry, Princeton University, Princeton, NJ 08544, USA; Department of Chemistry, Princeton University, Princeton, NJ 08544, USA

**Keywords:** Biocatalysis, Radical cyclization, Photoenzymatic reaction, Natural product synthesis, ene-reductase

## Abstract

Radical cyclizations are essential reactions in the biosynthesis of secondary metabolites and the chemical synthesis of societally valuable molecules. In this review, we highlight the general mechanisms utilized in biocatalytic radical cyclizations. We specifically highlight cytochrome P450 monooxygenases (P450s) involved in the biosynthesis of mycocyclosin and vancomycin, nonheme iron- and α-ketoglutarate-dependent dioxygenases (Fe/αKGDs) used in the biosynthesis of kainic acid, scopolamine, and isopenicillin N, and radical *S*-adenosylmethionine (SAM) enzymes that facilitate the biosynthesis of oxetanocin A, menaquinone, and F420. Beyond natural mechanisms, we also examine repurposed flavin-dependent “ene”-reductases (ERED) for non-natural radical cyclization. Overall, these general mechanisms underscore the opportunity for enzymes to augment and enhance the synthesis of complex molecules using radical mechanisms.

## Introduction

**Fig. 1.**
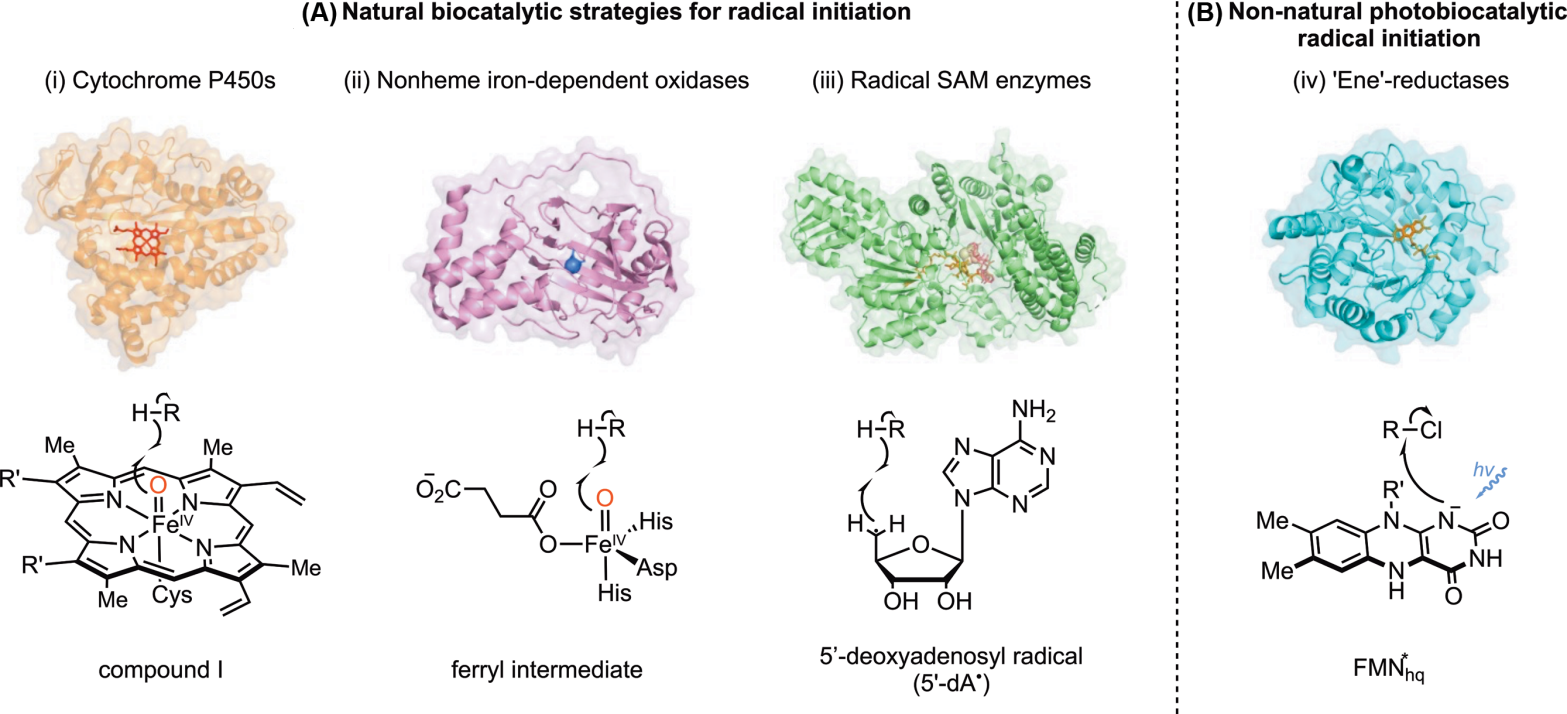
Biocatalytic strategies for radical initiation.

Radical cyclization reactions are ubiquitous in the synthesis of complex molecules (Corsello et al., 2017; Guo et al., 2019; Hung et al., 2018; Lu et al., 2018), leading to the development of numerous generalizable strategies for initiating and terminating radical intermediates (Clark, 2016; Ishibashi, 2006; Majumdar et al., 2007; Sibi et al., 2003; Tucker et al., 2010). While the reactivity patterns of these intermediates are well understood and predictable, controlling the stereochemical outcome of radical cyclizations remains a challenge for traditional small-molecule catalysts (Ishibashi, 2006; Sibi et al., 2003). Nature has evolved enzymes to catalyze these transformations in the synthesis of structurally complex natural products (Tang et al., 2017; Walsh & Moore, 2019; Walsh & Tang, 2018). While radicals follow similar reactivity patterns in enzyme active sites, they are formed using mechanisms that are distinct from those commonly used by small-molecule catalysts. Three major families of biocatalysts are known to catalyze radical cyclizations: (i) cytochrome P450 monooxygenases (P450s), (ii) nonheme iron- and α-ketoglutarate-dependent dioxygenases (Fe/αKGDs), and (iii) radical *S*-adenosylmethionine (SAM) enzymes (Fig. 1A) (Tang et al., 2017; Walsh & Moore, 2019). From the perspective of reaction mechanism, P450s and Fe/αKGDs both use high-valent oxo-iron species (compound I and ferryl intermediate, respectively) to oxidatively initiate radical formation via hydrogen atom transfer (Denisov et al., 2005; Martinez & Hausinger, 2015) while radical SAM enzymes use a 5′-adenosyl radical intermediate (5′-dA^•^) to initiate hydrogen atom transfer (Fig. 1A) (Nicolet, 2020; Ruszczycky et al., 2018; Yokoyama & Lilla, 2018). In chemical synthesis, dehalogenation is often used to form radical intermediates. Recently, enzymatic catalysts have been developed that take advantage of this mechanism of radical formation. Specifically, flavin-dependent “ene”-reductases (EREDs) were demonstrated to catalyze non-natural stereoselective radical cyclizations under visible light, where the excited state of cofactor flavin hydroquinone (FMN_hq_*) is responsible for the reductive radical initiation via single-electron transfer (Fig. 1B) (Beigasiewicz et al., 2019; Hyster, 2020). In this review, we summarize radical cyclization processes by discussing representative examples of involving P450s, Fe/αKGDs, SAM enzymes, and EREDs.

## Cytochromes P450-Catalyzed Radical Cyclization Reaction

### General Mechanism

Cytochrome P450 monooxygenases (P450s) are a large superfamily of iron and heme-dependent enzymes that catalyze oxidative transformations of a variety of endogenous and exogenous substrates, including xenobiotic metabolism and biosynthesis of steroids, lipids, vitamins, and natural products (Cochrane & Vederas, 2014; Denisov et al., 2005; Rudolf et al., 2017; Urlacher & Girhard, 2019; Whitehouse et al., 2012). P450s are best-known to catalyze hydroxylation reactions: the insertion of a single oxygen atom into a C–H bond of a substrate. In addition, they facilitate a diverse array of other reactions, including epoxidation, *N, S*-oxidation, *N*-, *O*-, *S*-dealkylation, C–C bond cleavage, and Baeyer–Villiger-type oxidation. Most recently, they have been employed to catalyze non-natural C–C and C–N bond-forming reactions via carbene and nitrene transfer reactions, making them one of the most favorable enzyme families for chemical synthesis (Bernhardt & Urlacher, 2014; Chen & Arnold, 2020; Fasan, 2012; Wei et al., 2018).

**Scheme 1.**
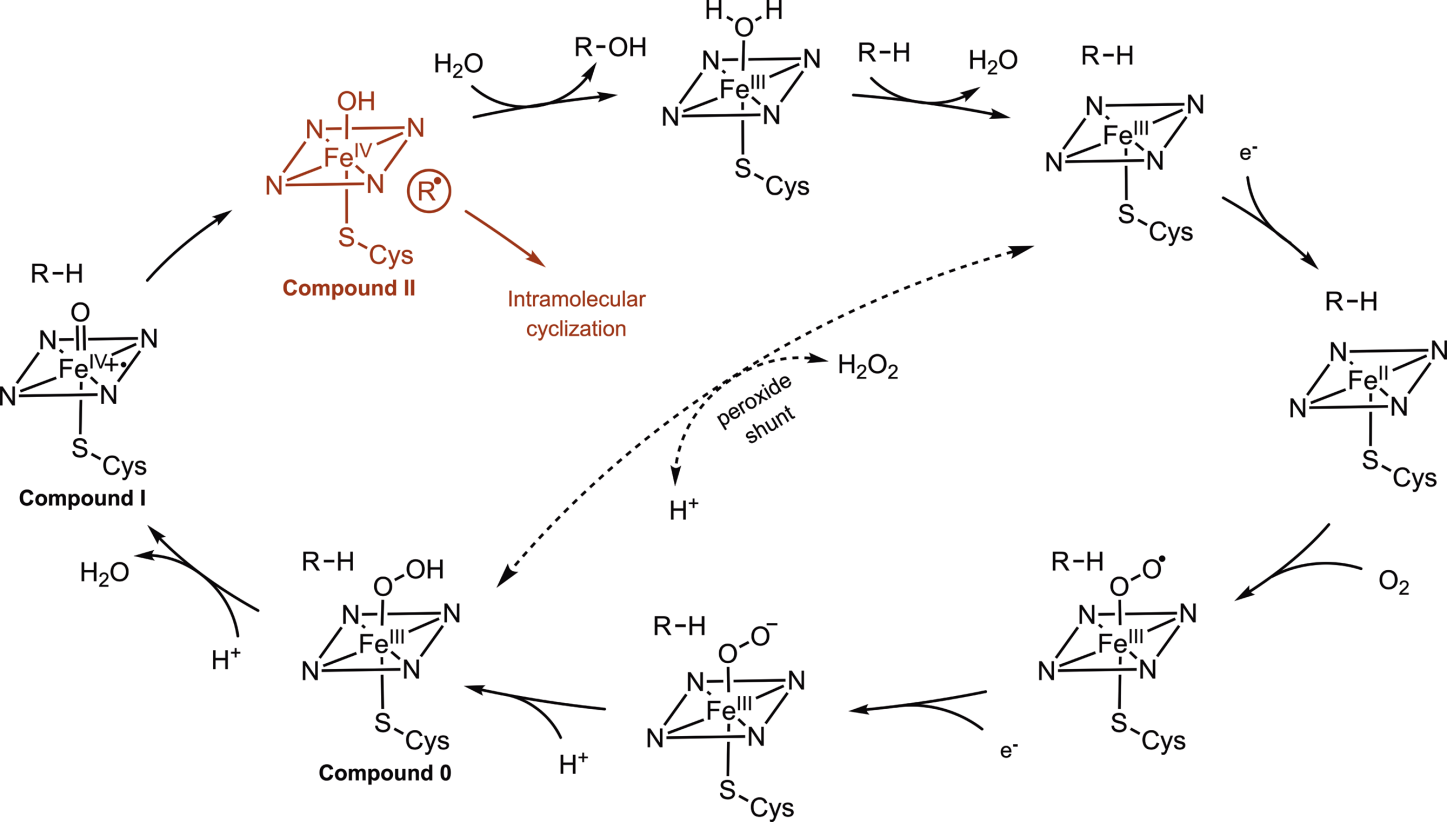
Catalytic cycle of cytochromes P450. R–H stands for the substrate. R–OH is the resulting hydroxylated product (some P450s can use the peroxide shunt pathway to directly produce Compound 0 from the substrate-bound high-spin Fe^III^ state using H_2_O_2_, bypassing the first three catalytic steps).

A simplified catalytic cycle of a P450s-catalyzed hydroxylation is shown in Scheme 1. The multistep catalytic cycle starts with binding of a substrate (R–H) to the enzyme active site that induces a spin shift of the ferric iron, allowing Fe^III^-to-Fe^II^ reduction by a first electron derived from NAD(P)H via the redox partners (Mclean et al., 2015). Binding of molecular oxygen to heme-Fe^II^ followed by a second electron transfer and protonation forms the hydroperoxy–ferric complex (Fe^III^–OOH, Compound 0). Protonation of the terminal oxygen and subsequently loss of a water leads to the formation of a high valent oxo-ferryl π-cation radical intermediate (Fe^IV^═O, Compound I) (Rittle & Green, 2010). Compound I can abstract a hydrogen atom from the substrate (R–H) to form a carbon-centered radical species (R^•^), which rapidly rebounds with the equivalent of hydroxyl radical (Fe^IV^−OH, Compound II) to generate the hydroxylated product (R–OH) (Ortiz de Montellano, 2010). Release of the product and re-coordination by water regenerates the ferric resting state of the catalyst. In addition to C–H bonds, Compound I can abstract a hydrogen atom from the O–H bond of phenols or the N–H bond of aniline to yield oxygen-centered or nitrogen-centered radicals (Denisov et al., 2005; Whitehouse et al., 2012). Notably, for certain P450 biocatalysts, the radical rebound step is slower than intramolecular radical rearrangement or diradical recombination (e.g., cyclization, ring expansion and fusion) (red part in Scheme 1) (Guengerich & Yoshimoto, 2018; Tang et al., 2017), allowing the formation of complexity-added products without incorporation of any oxygen atom (Tang et al., 2017; Walsh & Moore, 2019; Walsh & Tang, 2018).

### P450-Catalyzed Biosynthesis of Mycocyclosin (**3**)

**Scheme 2.**
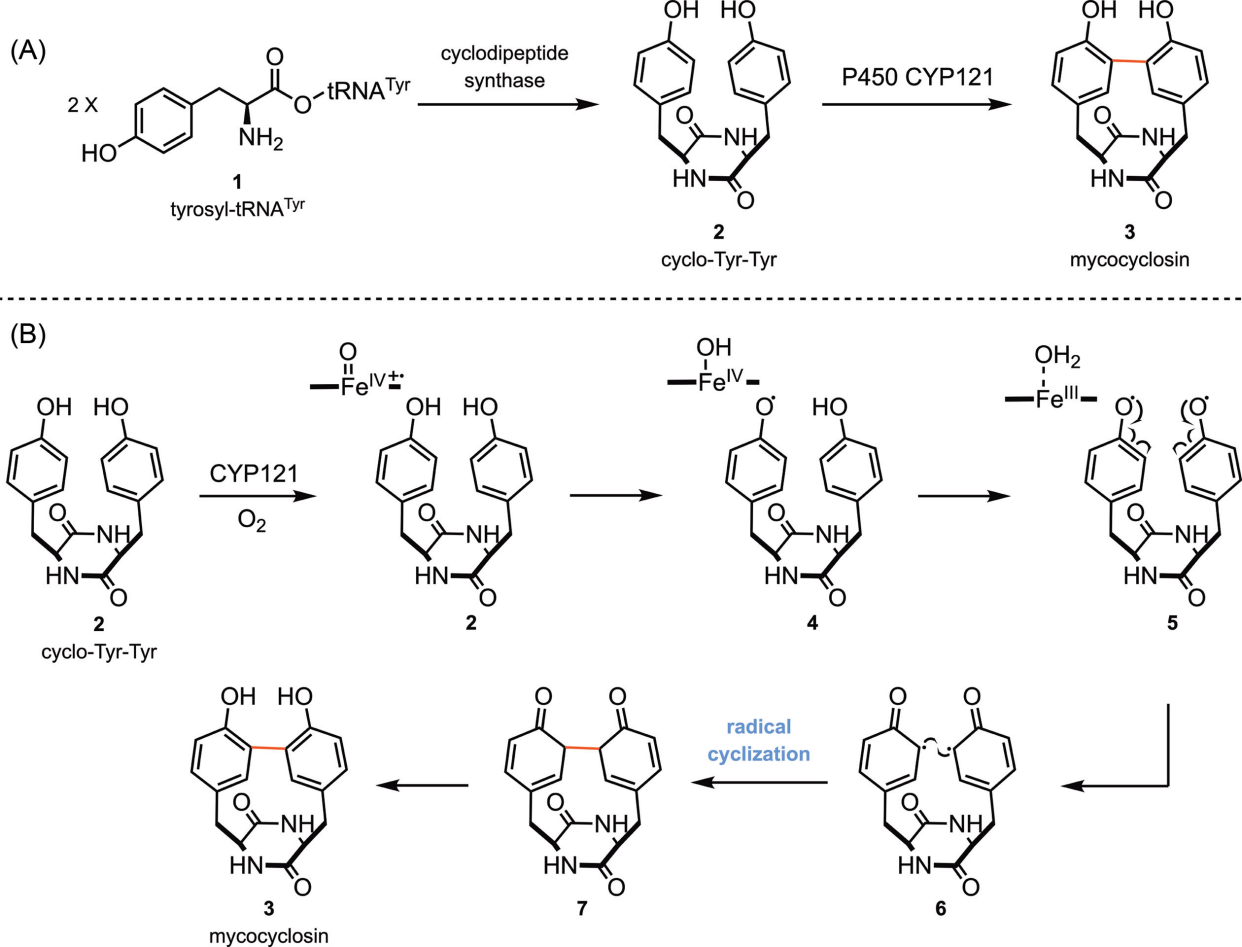
(A) Biosynthesis of mycocyclosin. (B) Proposed mechanism of the intramolecular diradical cyclization catalyzed by P450 CYP121.

The biosynthesis of mycocyclosin (**3**) is a representative example of a P450-catalyzed radical cyclization (Belin et al., 2009). Two sequential genes are responsible for the biosynthesis of mycocyclosin. The first enzyme is a cyclodipeptide synthase (Rv2275), which catalyzes the dipeptide bond formation of cyclo-Tyr-Tyr (**2**) using two molecules of tyrosyl-tRNA^Tyr^ (**1**) as substrates (Vetting et al., 2010). Subsequent coupling of the phenol rings of cyclo-Tyr-Tyr (**2**) is catalyzed by cytochrome P450 CYP121 (Rv2276) to provide mycocyclosin (**3**, Scheme 2A) (Belin et al., 2009). A diradical combination mechanism is proposed for the intramolecular cyclization step (Scheme 2B) (Belin et al., 2009). Mechanistically, this occurs via formation of Compound I (Fe^IV^═O, Scheme 1), which abstracts a phenolic hydrogen atom from **2** to form the tyrosyl radical intermediate **4** and Compound II (Fe^IV^–OH). Subsequently, a second phenolic hydrogen atom of **4** is abstracted by the resulting Compound II to yield the *O*-diradical 5, which isomerizes to provide C-diradical **6**. The following intramolecular diradical combination of C-diradical **6** forges the new C–C bond **7** and gives the final product mycocyclosin (**3**) after rearomatization (Belin et al., 2009; Dornevil et al., 2017; Dumas et al., 2014). Notably, P450 CYP121 is also found to be essential for *M. tuberculosis* growth by *in vitro* gene knockout studies (Mclean et al., 2008), which makes it an intriguing therapeutic target for antituberculosis (Kishk et al., 2019).

### P450s-Catalyzed Biosynthesis of Vancomycin (**13**)

**Scheme 3.**
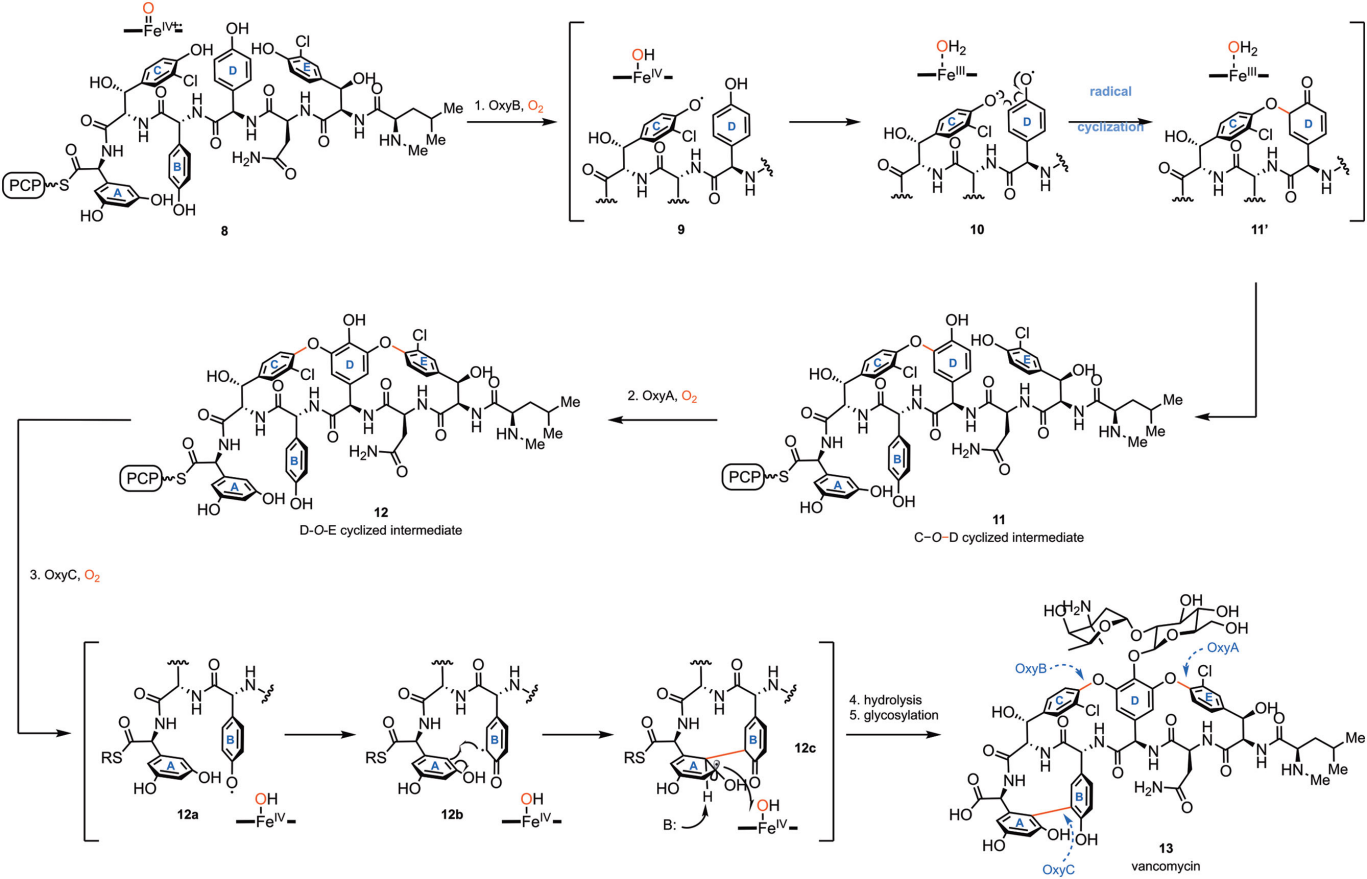
Biosynthesis of vancomycin (**13**) and the proposed radical mechanism of the intramolecular cyclizations catalyzed by P450 enzymes (OxyA/B/C).

Glycopeptide antibiotics (GPAs), such as vancomycin (**13**), have been used clinically to treat serious infections caused by Gram-positive bacteria for decades (Kahne et al., 2005; Levine, 2006; Yim et al., 2014). Structurally, vancomycin (**13**) is a strained and atropisomeric heptapeptide with two biaryl ether (C−*O*−D and D−*O*−E) and one biaryl (A−B) crosslinks between the side chains of aromatic amino acids (Scheme 3). The linear heptapeptide backbone (**8**) is assembled by nonribosomal peptide synthetase (NRPS) and contains Asn, Leu, and five nonproteinogenic aromatic amino acids (Haslinger et al., 2015; Peschke et al., 2016; Yim et al., 2014). The aromatic side-chain crosslinking of the heptapeptide precursor **8** is catalyzed by three P450 enzymes (OxyA, OxyB, and OxyC) with a strict order, featuring multiple C−O and C−C coupling steps (Bischoff et al., 2001; Stegmann et al., 2006): OxyB catalyzes the biaryl ether C−*O*−D cross-coupling (C−O coupling) first (Woithe et al., 2007; Zerbe et al., 2004); OxyA then catalyzes the D−*O*−E biaryl ether cross-coupling (C−O coupling) (Forneris et al., 2017); finally OxyC catalyzes the biaryl A−B crosslinking (C−C coupling) (Forneris & Seyedsayamdost, 2018). After the sequential three steps of cyclization, the resulting cyclic vancomycin aglycone is released from the NRPS assembly via hydrolysis by the thioesterase domain and is further glycosylated to provide the final glycopeptide vancomycin (Scheme 3) (Yim et al., 2014).

Diradical combination mechanisms were proposed for the cyclization reactions by structural and mechanistic studies (Scheme 3) (Pylypenko et al., 2003; Tang et al., 2017; Walsh & Tang, 2018; Woithe et al., 2007; Zerbe et al., 2002). As shown in Scheme 3, the OxyB-catalyzed C−*O*−D cyclization starts with the abstraction of a phenolic hydrogen atom from the C-ring of **8** by Compound I, yielding a single radical intermediate **9** and Compound II. Subsequently, Compound II abstracts a phenolic hydrogen atom from the D-ring of **9** to give the *O*-diradical **10**. Delocalization of the phenoxy radical on D-ring to its *ortho* carbon followed by diradical combination forges the new C–O bond between the C and D ring in **11**′, providing **11** after tautomerization. The mechanism of the A−B cyclization process catalyzed by OxyC is similar to the one involved in the biosynthesis of mycocyclosin (Scheme 2B). Recently, an alternative mechanism involving a single radical intermediate was also proposed for the OxyC (Scheme 3) (Forneris & Seyedsayamdost, 2018).

In addition to the two examples discussed above, other examples of P450s-catalyzed intramolecular radical cyclizations can be found in the biosynthesis of indolocarbazole alkaloids staurosporine and rebeccamycin (C–C coupling) (Howard-Jones & Walsh, 2007; Makino et al., 2007), glycopeptide teicoplanin (C–O and C–C coupling) (Li et al., 2011), spirocyclic griseofulvin (C–O coupling) (Grandner et al., 2016), alkaloid salutaridine (C–C coupling) (Gesell et al., 2009), and fumitremorgin (C–N coupling) (Kato et al., 2009).

## Nonheme Iron Oxidase-Catalyzed Radical Cyclization Reactions

### General Mechanism

**Scheme 4.**
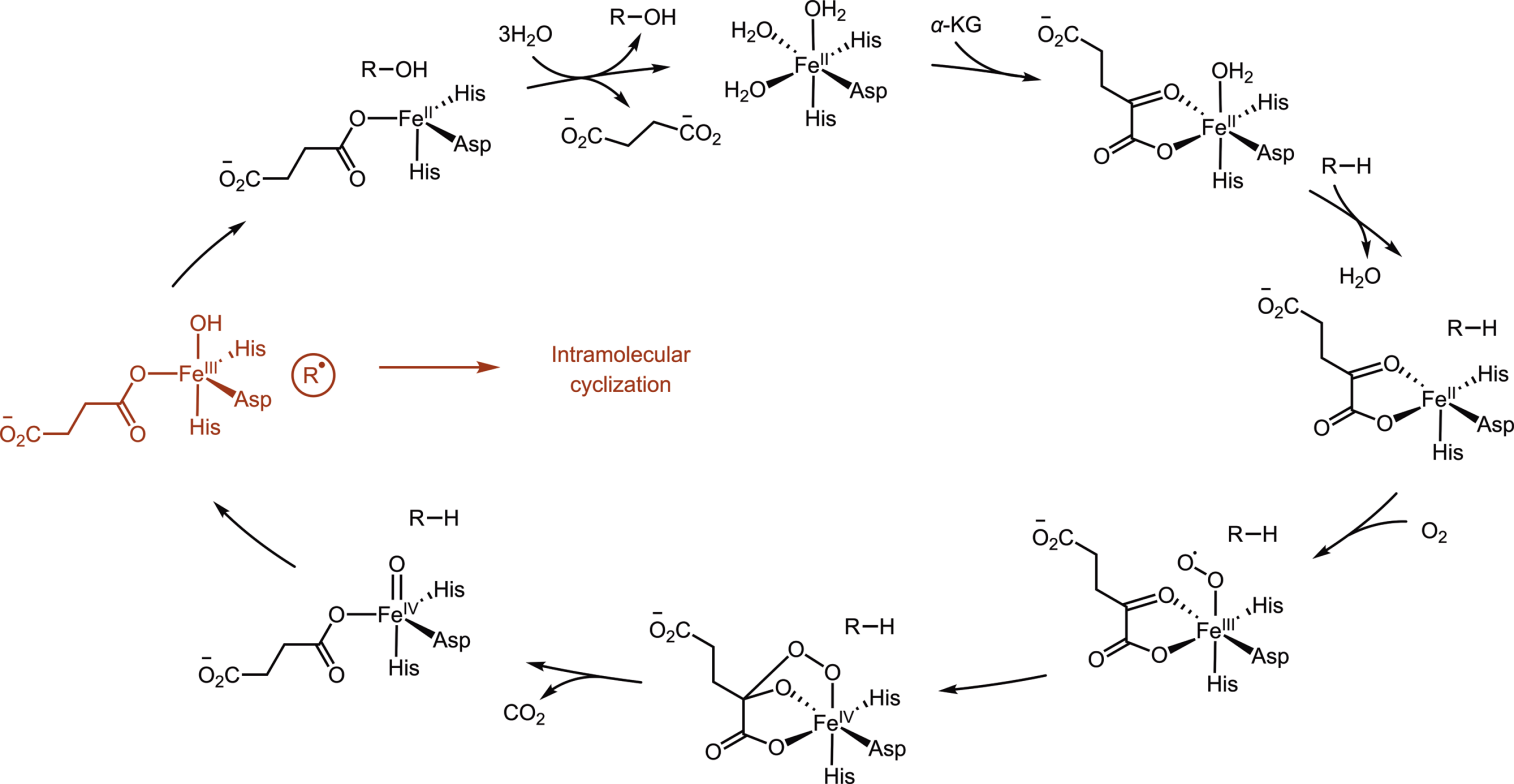
Catalytic cycle of Fe/αKGDs-catalyzed hydroxylation.

The mononuclear nonheme iron- and α-ketoglutarate (αKG)-dependent dioxygenases (Fe/αKGDs) require iron (II) as metallocofactor and α-KG as co-substrate (Hausinger, 2015; Herr & Hausinger, 2018; Loenarz & Schofield, 2008). Structurally, Fe/αKGD enzymes share a conserved double-stranded β-helix (DSBH) fold that coordinates the Fe center with two histidine residues and one carboxylate from either a glutamic acid or an aspartic acid residue (2-His-1-carboxylate facial triad) (Hegg & Que, 1997). Fe/αKGDs catalyze a variety of oxidative reactions, including hydroxylation, halogenation, cyclization, desaturation, epimerization, C–C bond cleavage, and epoxidation, as such playing an important role in the biosynthesis of secondary metabolites (Gao et al., 2018; Hausinger, 2015; Herr & Hausinger, 2018; Krebs et al., 2007; Loenarz & Schofield, 2008, 2011; Wu et al., 2016; Zwick & Renata, 2020). The putative mechanism of Fe/αKGDs-catalyzed hydroxylation was shown in Scheme 4 (Hausinger 2015; Krebs et al., 2007; Martinez & Hausinger, 2015). The catalytic cycle starts with the binding of co-substrate α-KG to the Fe^II^ center, during which two of the three metal-bound water molecules are replaced. Upon binding of the primary substrate (R–H) to the enzyme active site, the third metal-bound water is removed, allowing the binding of molecular oxygen to form a Fe^III^-superoxo intermediate. The distal oxygen atom of the Fe^III^-superoxo species attacks C2 of α-KG to yield a peroxohemiketal bicyclic intermediate, followed by oxidative decarboxylation to release CO_2_ and provide a Fe^IV^-oxo species (also termed as the ferryl intermediate). Like Compound I in P450s-catalyzed hydroxylation, this ferryl species (Fe^IV^═O) abstracts a hydrogen atom from the primary substrate (R–H) to generate a radical intermediate (R^•^) and the Fe^III^–OH species. The radical (R^•^) can rebound with the hydroxyl radical to give the final product (R–OH), with concomitant formation of Fe^II^. After the release of the hydroxylated product (R–OH) and succinate, the resting state (Fe^II^) of Fe/αKGD is regenerated by re-coordinating with three water molecules, thus completing the catalytic cycle (Hausinger 2015; Martinez & Hausinger 2015). Notably, in some cases, instead of the hydroxyl radical rebound step, the substrate centered radical (R^•^) can undergo a competing intramolecular radical cyclization to form the cyclic products (red part in Scheme 4) (Tang et al., 2017; Walsh & Moore, 2019; Walsh & Tang, 2018).

### Fe/αKGD-Catalyzed Biosynthesis of Kainic Acid (**17**)

**Scheme 5.**
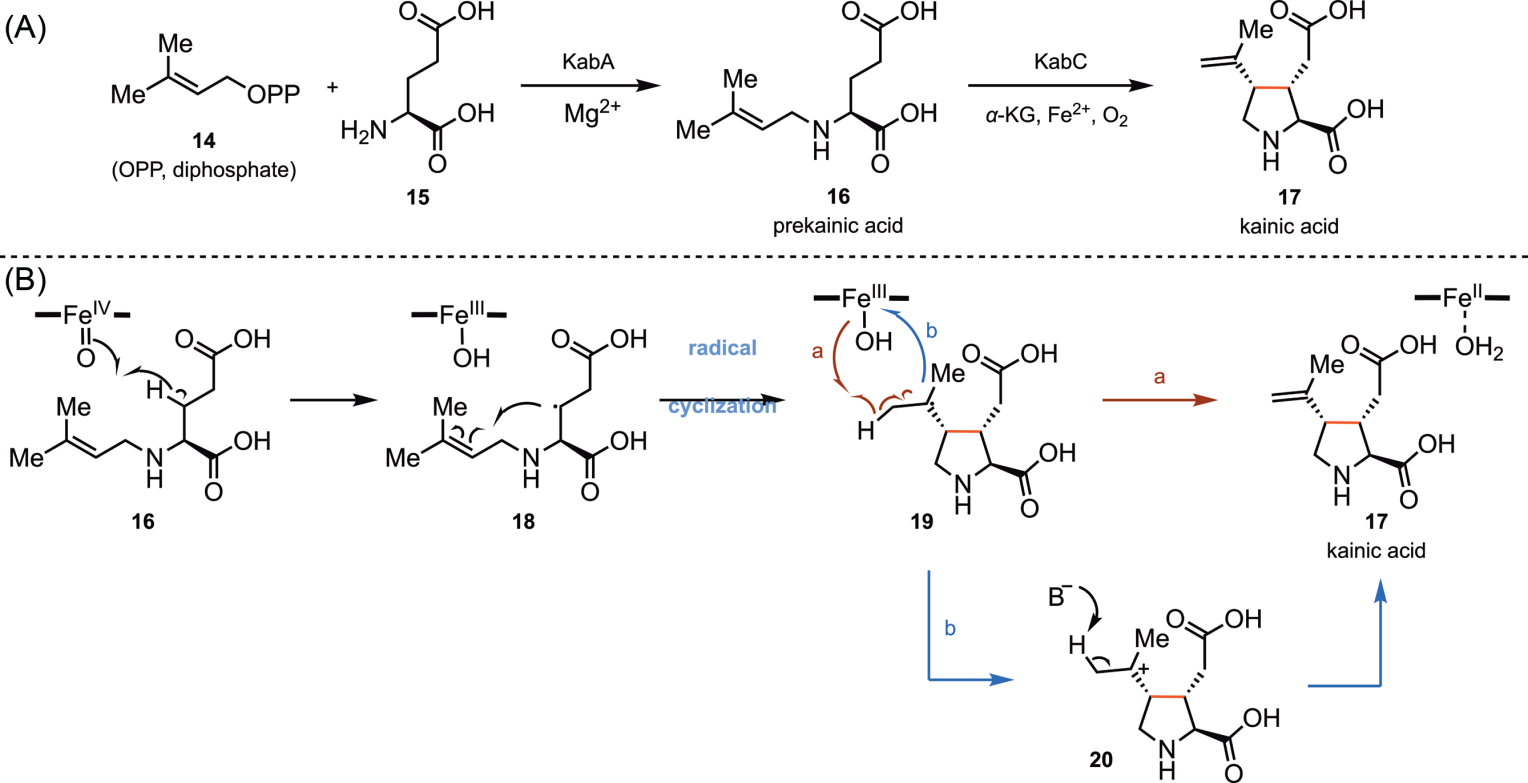
(A) Biosynthesis of kainic acid (**17**) catalyzed by KabA and KabC. (B) The proposed radical mechanism of KabC-catalyzed cyclization.

Kainic acid (KA, **17**, Scheme 5) is a member of kainoids natural product (pyrrolidinedicarboxylic acids), and it was first isolated from the tropical seaweed *Digenea simplex*, which has been used for the treatment of *Ascaris* infections for centuries in Asia (Higa & Kuniyoshi, 2000). Kainic acid (**17**), a cyclic analog of l-glutamic acid, was identified as a potent ionotropic glutamate receptor (iGluR) agonist and serves as an important pharmacological tool in many neurophysiological studies (Lodge, 2009; Werner et al., 1991; Zheng et al., 2011). Since its discovery in the 1950s, the interesting structural features and important biological activities of kainic acid have attracted the attention of synthetic chemists, leading to the development of numerous synthetic routes (Stathakis et al., 2012). Recently, a concise two-enzyme (KabA and KabC) biosynthetic pathway was reported by Moore and coworkers (Chekan et al., 2019). Specifically, *N*-prenylation of l-glutamic acid (**15**) with dimethylallyl pyrophosphate (**14**) is catalyzed by a *N*-prenyltransferase KabA to provide the prekainic acid (**16**), which then undergoes an oxidative cyclization catalyzed by a Fe/αKGD enzyme KabC to form the final product kainic acid (**17**, Scheme 5A) (Chekan et al., 2019). As shown in Scheme 5B, a mechanism involving radical cyclization was proposed for the KabC-catalyzed C–C bond forming step (Chekan et al., 2019). First, the resting state of the KabC reacts with co-substrate α-KG and O_2_ to form the ferryl species (Fe^IV^═O). The ferryl species then abstracts a β-hydrogen atom from **16**, leading to the formation of radical intermediate **18** and the Fe^III^–OH species. Radical **18** can undergo cyclization to form the pyrrolidine ring in intermediate **19**. Finally, kainic acid (**17**) is generated via either a hydrogen atom transfer pathway (route a, Scheme 5B) or an oxidation/deprotonation pathway (route b, Scheme 5B) (Chekan et al., 2019; Dunham et al., 2018).

### Fe/αKGD-Catalyzed Biosynthesis of Scopolamine (**23**)

**Scheme 6.**
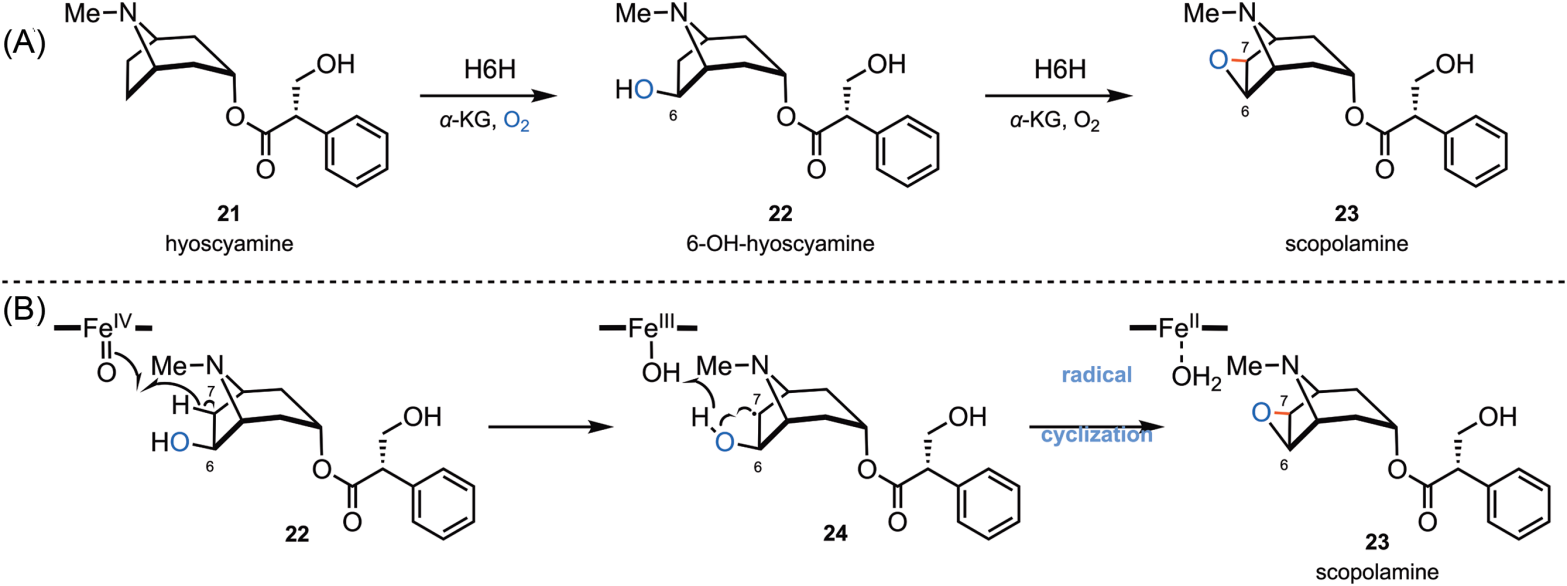
(A) Biosynthetic pathway from hyoscyamine (**21**) to scopolamine (**23**) catalyzed by a Fe/αKGD enzyme hyoscyamine 6β-hydroxylase (H6H). (B) The proposed radical mechanism for the second cyclization step catalyzed by H6H.

Scopolamine (**23**, Scheme 6), also known as hyoscine, is a tropane alkaloid found in Solanaceae plants (Hashimoto & Yamada, 1986; Matsuda et al., 1991), It has been used to treat motion sickness, postoperative nausea, and vomiting for decades (Clissold & Heel, 1985). Hyoscyamine (**21**), the key biosynthetic precursor of scopolamine (**23**), is formed by the condensation of tropine and tropic acid, which are originated from l-ornithine and l-phenylalanine, respectively (Hashimoto & Yamada, 1986, 1987; Zhang et al., 2004). As shown in Scheme 6A, hyoscyamine (**21**) is converted to scopolamine (**23**) via 6-hydroxyhyoscyamine (**22**) by two successive oxidation steps catalyzed by hyoscyamine 6β-hydroxylase (H6H, a Fe/αKGD enzyme) (Hashimoto & Yamada, 1986, 1987; Zhang et al., 2004). 6-Hydroxyhyoscyamine (**22**) is generated from C6-hydroxylation of hyoscyamine (**21**) catalyzed by H6H through hydrogen atom abstraction followed by hydroxyl radical rebound. In the second step, epoxidation of **22** to form the final product scopolamine (**23**) is also catalyzed by H6H, through a radical cyclization mechanism (Scheme 6B). The ferryl species (Fe^IV^═O) abstracts one hydrogen atom from C7 of **22**, leading to a C-radical intermediate **24**. The resulting Fe^III^–OH species can abstract one hydrogen atom from the hydroxyl group at C6 of **24**, facilitating epoxide formation via radical/radical C−O coupling to yield the final product scopolamine (**23**, Scheme 6B) (Tang et al., 2017). In addition to the examples discussed before, Fe/αKGDs-catalyzed intramolecular radical cyclizations can also be found in the biosynthesis of β-lactam clavaminic acid (C−O coupling) (Borowski et al., 2007; Hamed et al., 2013), endoperoxide verruculogen (C−O coupling) (Yan et al., 2015), oligosaccharide orthosomycin (C−O coupling) (McCulloch et al., 2015), and lolines (Pan et al., 2018).

### Nonheme, Iron-Dependent Oxidase in the Biosynthesis of Isopenicillin N (**28**)

**Scheme 7.**
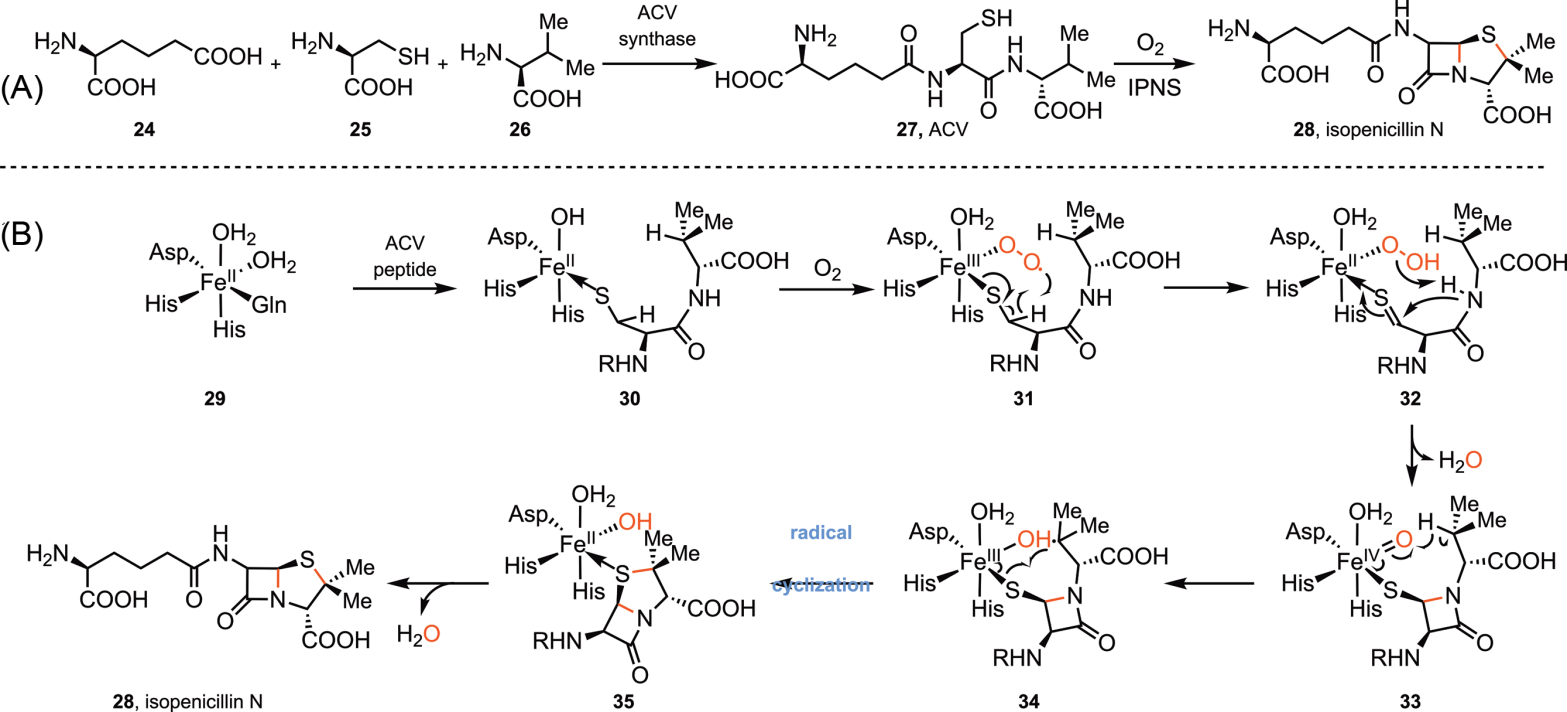
(A) Biosynthesis of isopenicillin N (**28**) and (B) the proposed radical mechanism by isopenicillin N synthase (IPNS).

In addition to the P450 and Fe/αKGD enzymes, there are other iron-dependent enzymes that facilitate radical cyclization reactions (Sydor et al., 2011). A representative example is Isopenicillin N synthase (IPNS). IPNS is a nonheme iron-dependent oxidase that involves in the two-step biosynthesis of isopenicillin N (**28**, Scheme 7), a key precursor for the well-known β-lactam antibiotics penicillins and cephalosporins (Hamed et al., 2013; Ozcengiz & Demain, 2013; Rabe et al., 2018; Roach et al., 1997; Townsend, 2016). As shown in Scheme 7, the linear tripeptide precursor δ-(l-α-aminoadipoyl)-l-(cysteinyl)-d-valine (**27**, ACV) is biosynthesized by the ACV synthetase, a NRPS that condenses l-α-aminoadipic acid (**24**), l-cysteine (**25**), and l-valine (**26**, the stereochemistry is inverted during peptide formation) (Byford et al., 1997). The linear ACV tripeptide (**27**) is then oxidatively cyclized to form the fused β-lactam and thiazolidine core of isopenicillin N (**28**) by IPNS through a radical mechanism (Scheme 7B) (Roach et al., 1997). Overall, the IPNS-catalyzed oxidation proceeds in two phases, each involving a two-electron-oxidative cyclization, to give the 4-membered β-lactam ring and the 5-membered thiazolidine ring of isopenicillin N (**28**), successively. The cofactor α-KG is not needed (Scheme 7B) (Rabe et al., 2018; Roach et al., 1997; Tamanaha et al., 2016). The first β-lactam ring formation starts with the ligation of substrate ACV (**27**) to the IPSN iron center, followed by reaction with O_2_ to generate the Fe^III^-superoxo species (**31**). The Fe^III^-superoxo species can abstract a *pro*-(*S*) cysteinyl C-3 hydrogen atom to form a thioalkyl radical, yielding a Fe^II^-hydroperoxo intermediate and a thioaldehyde after an inner-sphere electron transfer (**32**). Heterolysis of the peroxo bond with concomitant deprotonation of the valinyl amido hydrogen allows the nucleophilic attack of the amide nitrogen onto the thioaldehyde, forming a monocyclic β-lactam intermediate coordinated to the Fe^IV^-oxo (ferryl) species (**33**) (Burziaff et al., 1999). In the second phase, the ferryl species (**33**) abstracts the valine C-3 hydrogen atom, resulting in a ferric hydroxyl (Fe^III^–OH) species and a valinyl radical (**34**). The valinyl radical then attacks the coordinated sulfur atom to provide the thiazolidine ring of the final product isopenicillin N (**28**) and restore the metal to the resting Fe^II^ state (Scheme 7B) (Tamanaha et al., 2016).

## *S*-Adenosylmethionine-Dependent Enzymes Catalyzed Radical Cyclization Reactions

### General Mechanism

**Scheme 8.**
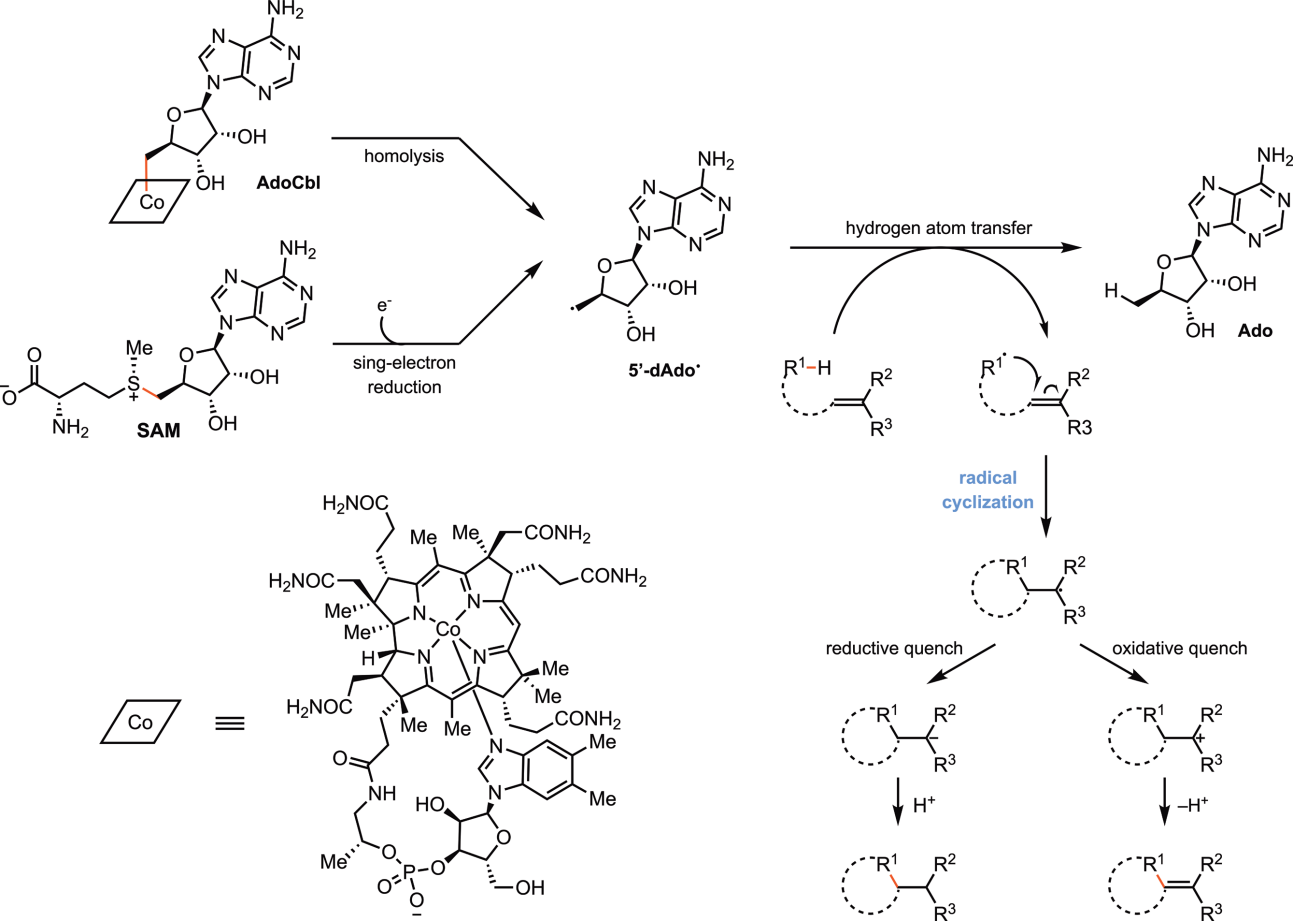
General mechanism of radical cyclization reaction catalyzed by 5′-deoxyadenosyl radical (5′-dAdo**·**).

In addition to iron-dependent enzymes, nature has evolved another elegant approach to perform radical cyclization reactions using a highly reactive 5′-deoxyadenosyl radical (5′-dAdo**·**) (Scheme 8). A small group of adenosylcobalamin (AdoCbl)-dependent enzymes (Brown, 2005; Matthews, 2009) and a more recently recognized but much larger class of radical SAM enzymes (Broderick et al., 2014; Brown, 2005; Challand et al., 2011; Frey & Booker, 2001; Marsh & Román Meléndez, 2012; Matthews, 2009) are both able to initiate radical reactions by abstracting a hydrogen atom from a C–H bond of the substrate. The general mechanisms of radical cyclization reactions catalyzed by these two classes of enzymes are shown in Scheme 8. The Co–C bond in AdoCbl has a relatively low bond dissociation energy (BDE) of ∼30 kcal/mol (Yokoyama & Lilla, 2018) and the corresponding 5′-dAdo**·** is usually generated by direct homolytic cleavage. In contrast, the BDE of the C–S bond in SAM is much higher (∼60 kcal/mol) (Yokoyama & Lilla, 2018). As a result, the corresponding 5′-dAdo**·** could only be formed through a single-electron reduction, commonly facilitated by a reduced iron-sulfur cluster. The 5′-dAdo**·** formed in the enzyme active site can engage in a hydrogen atom transfer (HAT) with an enzyme-bound substrate (R–H) to yield 5′-deoxyadenosine (Ado) and a radical intermediate (R**·**) which can undergo a cyclization reaction. Such reactions catalyzed by radical SAM enzymes are independent of molecular oxygen and can be either oxidative or reductive quenched, forging a new C_sp3_–C_sp3_ or C_sp3_–C_sp2_ bond, respectively. The BDE for the 5′-dAdo**·** C5′–H bond is 94–101 kcal/mol (Luo, 2003), which is higher than sp^3^ (*t*-butyl C–H BDE: 96.5 kcal/mol) or activated sp^2^ (benzylic C–H BDE: 90 kcal/mol, C–H bond α to ether: 92 kcal/mol) C–H bonds. This renders these enzymes as powerful catalysts in biosynthetic pathways to install C–C bonds in unconventional positions and provide natural products and cofactors with great structural diversity.

### Cobalamin and SAM-Containing Enzymes in the Biosynthesis of Oxetanocin A

**Scheme 9.**
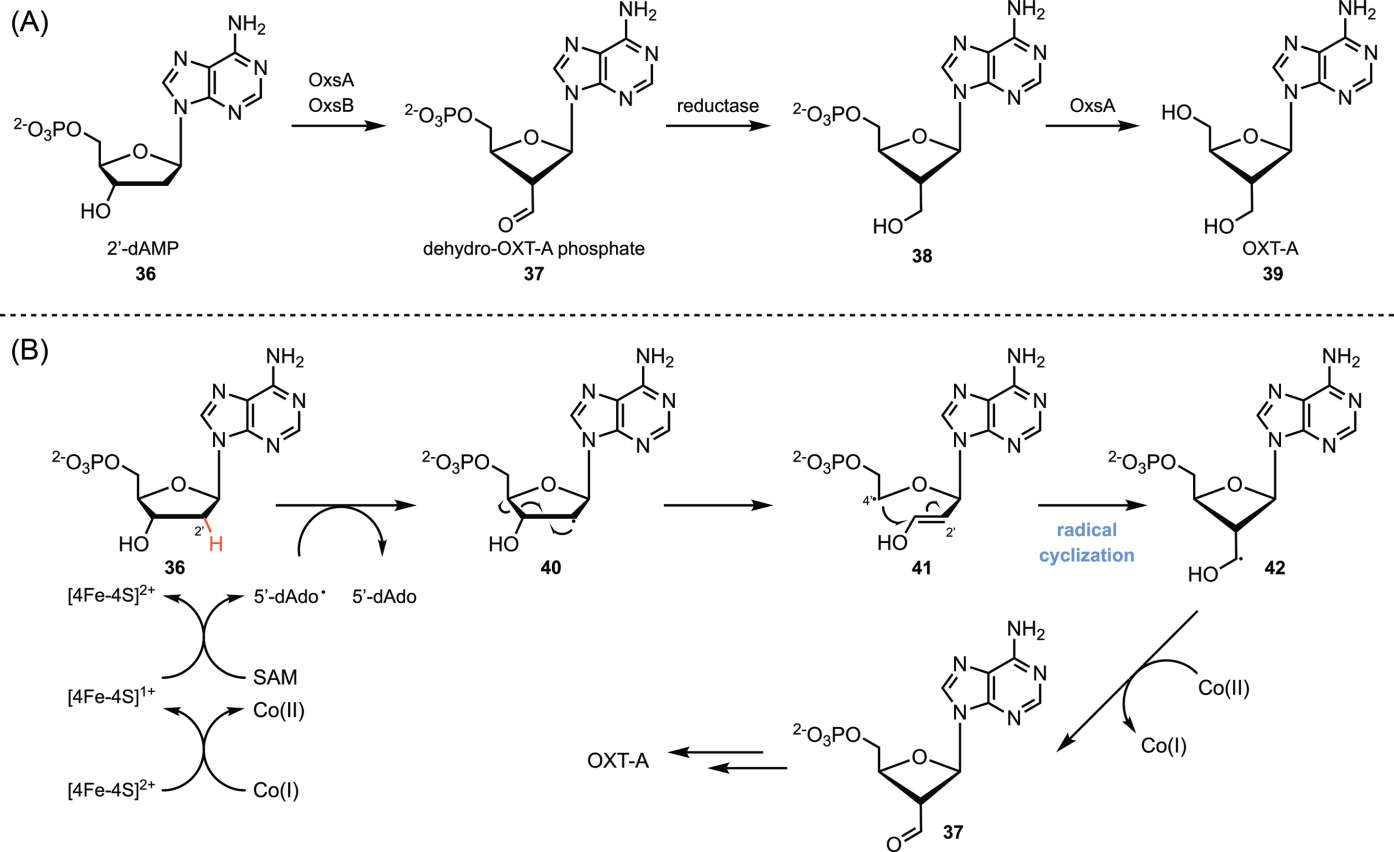
(A) Biosynthesis of OXT-A. (B) Proposed mechanism of the radical cyclization process catalyzed by OxsA and OxsB.

Enzymes only dependent on a Cobalamin cofactor catalyze various radical rearrangements (Banerjee, 2003; Wolthers et al., 2008) or elimination reactions (Sandala et al., 2006; Wetmore et al., 2002), but rarely radical cyclizations. Enzymes that rely on both cobalamin and SAM cofactors facilitate radical cyclizations in complex molecule biosynthesis. For instance, in the biosynthesis of oxetanocin A (OXT-A), an antiviral with a unique four-membered ring structure (Nakamura et al., 1986; Shimada et al., 1986), a cobalamin-dependent radical SAM enzyme OxsB is proposed to catalyze the key ring contraction step from 2′-dAMP (**36**) to dehydro-OXT-A phosphate (**37**) (Scheme 9) (Bridwell-Rabb et al., 2017). The reaction initiates by single-electron shuttling from Co(I) to the iron-sulfur cluster [4Fe-4S]^2+^, which generates Co(II) and [4Fe-4S]^+^. Subsequent reduction of SAM by [4Fe-4S]^+^ forms the active 5′-dAdo**·**, which abstracts the H2′ in 2′-dAMP (**36**). **36** then undergoes C3′–C4′ bond cleavage, followed by a radical cyclization reaction in which the resulting C4′ radical attacks C2′ to form the four-member ring intermediate **42**. It is proposed that Co(II) could then act as an electron acceptor and oxidize **42** to provide **37**, which regenerates Co(I) and completes the catalytic cycle. OXT-A is subsequently formed after reduction and hydrolysis.

### SAM-Containing Enzymes in the Biosynthesis of Menaquinone

**Scheme 10.**
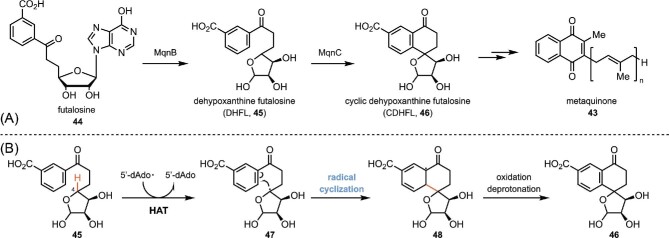
(A) Biosynthesis of metaquinone. (B) Proposed mechanism of the radical cyclization process catalyzed MqnC.

In terms of SAM-containing enzyme-catalyzed radical cyclization reactions, a representative example is the “futalosine pathway” biosynthesis of menaquinone (Hiratsuka et al., 2008). Menaquinone is a lipid-soluble small molecule that serves as an electron shuttle in the bacterial electron transport chain (Nowicka & Kruk, 2010) and also an essential vitamin in humans (vitamin K2), playing a critical role in blood coagulation (Cranenburg et al., 2007) and bone formation (Plaza & Lamson, 2005). The proposed biosynthetic pathway of menaquinone from futalosine (**44**) is shown in Scheme 10 (Cooper et al., 2013). Once formed, futalosine (**44**) is converted to dehypoxanthine futalosine (DHFL, **45**) by the hydrolase MqnB, followed by a radical cyclization that provides cyclic dehypoxanthine futalosine (CDHFL, **46**) by the radical SAM enzyme MqnC. The 5′dAdo**·** formed in MqnC abstracts the H4 in **45** through HAT and provides intermediate **47**. The resulting carbon-center radical attacks the phenyl ring at the position *para* to the carboxylate to form a C–C bond, generating intermediate **48**. Further oxidation and deprotonation of **48** afford CDHFL, which is the precursor of metaquinone.

### SAM-Containing Enzymes in the Biosynthesis of F420

**Scheme 11.**
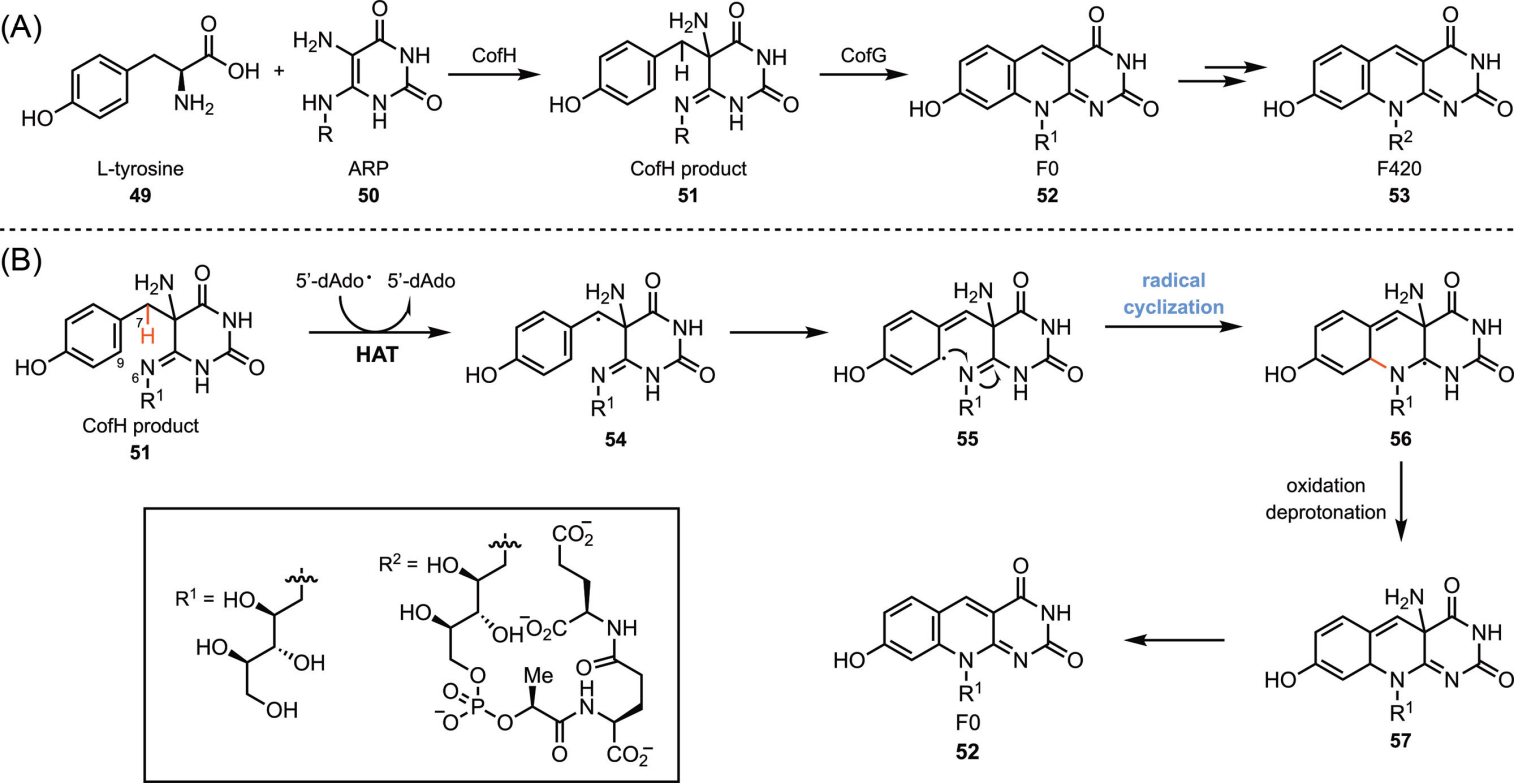
(A) Biosynthesis of F420. (B) Proposed mechanism of the radical cyclization process catalyzed CofG.

SAM-containing enzymes also catalyze radical C–N bond formation as in the case of F420 biosynthesis. F420 is a naturally occurring deazaflavin cofactor in which the N5 of the flavin ring is replaced with a methine. It functions as a potent two-electron reductant in cells (Walsh, 1986). The physiological functions of F420 and F420 dependent enzymes include anti-TB prodrug activation (Singh et al., 2008), resistant to oxidative stress (Purwantini & Mukhopadhyay, 2009), and biosynthesis of clinically important natural products (Coats et al., 1989; Nakano et al., 2004). The precursor of F420, F0 is formed by a reaction between 5-amino-6-ribitylamino-2,4-pyrimidinone (ARP) and tyrosine that is catalyzed by an F0 synthase (Decamps et al., 2012). In archaea and cyanobacteria, F0 synthase is encoded by two separate genes *cofG* and *cofH*. CofH is a radical SAM enzyme facilitating the formation of CofH product **51** from l-tyrosine and ARP (Scheme 11) (Philmus et al., 2015), Enzyme CofG is another radical SAM enzyme that catalyzes a radical cyclization reaction. Based on one of the proposed mechanism, CofG abstracts a hydrogen atom from the 7-position of **51** to form the C7 radical **54**. After tautomerization, the carbon-center radical in **55** attacks the imine N6 and form the C9–N6 bond. The resulting intermediate **56** is then oxidatively quenched and eliminates an ammonia to provide F0 (Mehta et al., 2015).

## Flavin-Dependent Enzymes Catalyzed Non-Natural Radical Cyclization Reactions

### Design of Biocatalytic Platform For Non-Natural Radical Cyclizations

**Scheme 12.**
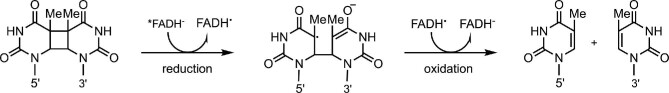
Mechanism of DNA photolyase.

Inspired by enantioselective radical cyclization reactions existing in nature, Hyster and coworkers sought to develop biocatalytic strategies to realize asymmetric cyclization reactions mediated by non-natural radical intermediates (Beigasiewicz et al., 2018, 2019; Clayman & Hyster, 2020; Black et al., 2019; Emmanuel et al., 2016; Hyster 2020; Nakano et al., 2019, 2020; Sandoval et al., 2017, 2019; Sandoval & Hyster, 2020). As many synthetic radical reactions are initiated via radical dehalogenation, they sought to develop mechanisms to carry out this fundamental mechanism. Inspired by the ability of flavin-dependent DNA photolyase to cleave weak bonds using single-electron reductions, Hyster and coworkers questioned whether substrate promiscuous enzymes would display the same reactivity patterns (Scheme 12) (Brettel & Byrdin, 2010). The group targeted EREDs (Heckenbichler et al., 2018; Toogood & Scrutton, 2018) as attractive scaffolds for the desired reactivity because of their ease of handling, substrate promiscuity, and evolvability render them one of the most ubiquitous families of enzymes in chemical synthesis.

### ERED-Catalyzed Reductive Radical Cyclization Reactions

**Scheme 13.**
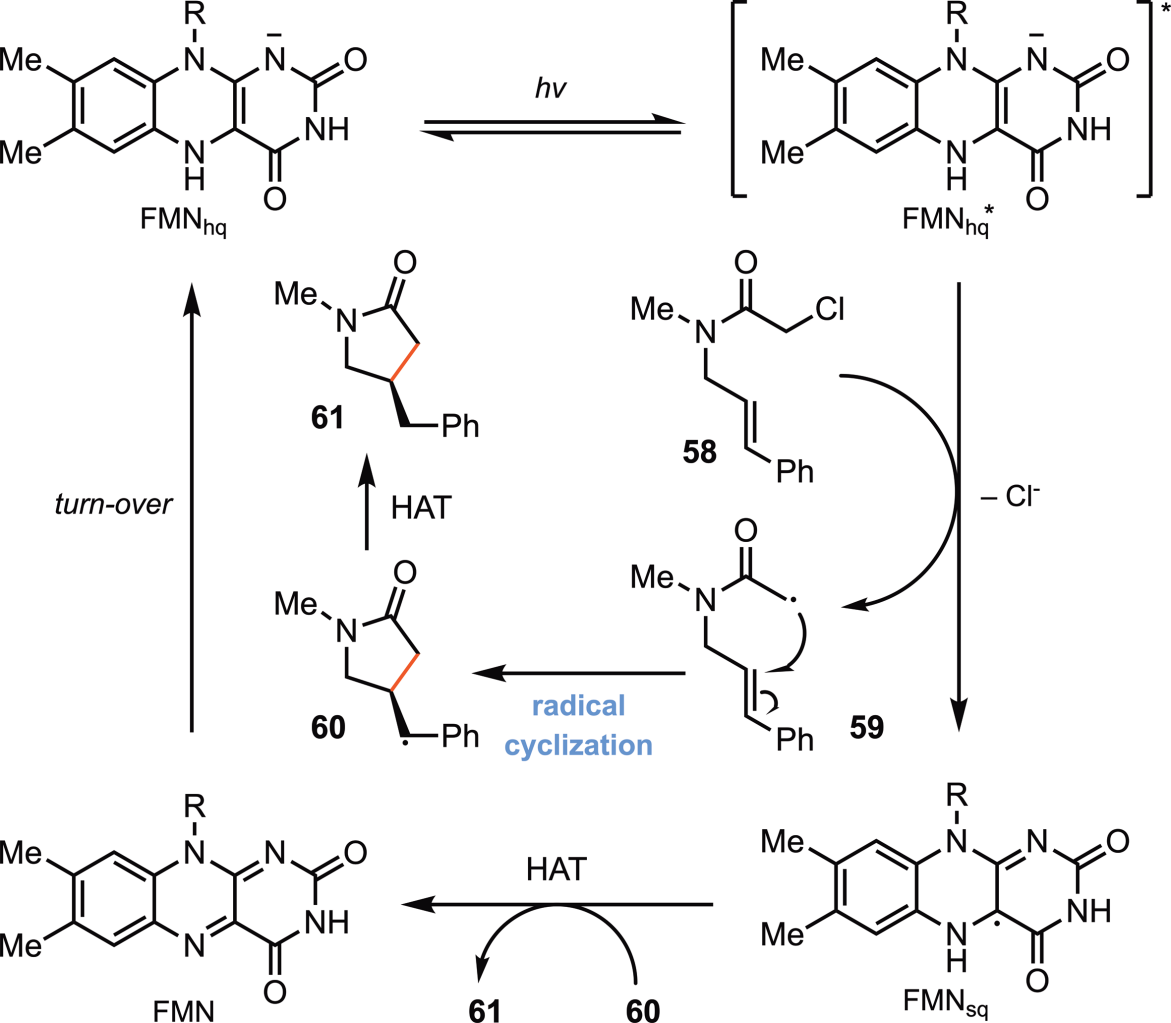
Proposed mechanism of the biocatalytic radical cyclization of α-chloroamides for the preparation of β-lactams catalyzed by photoexcited flavin proteins.

**Scheme 14.**
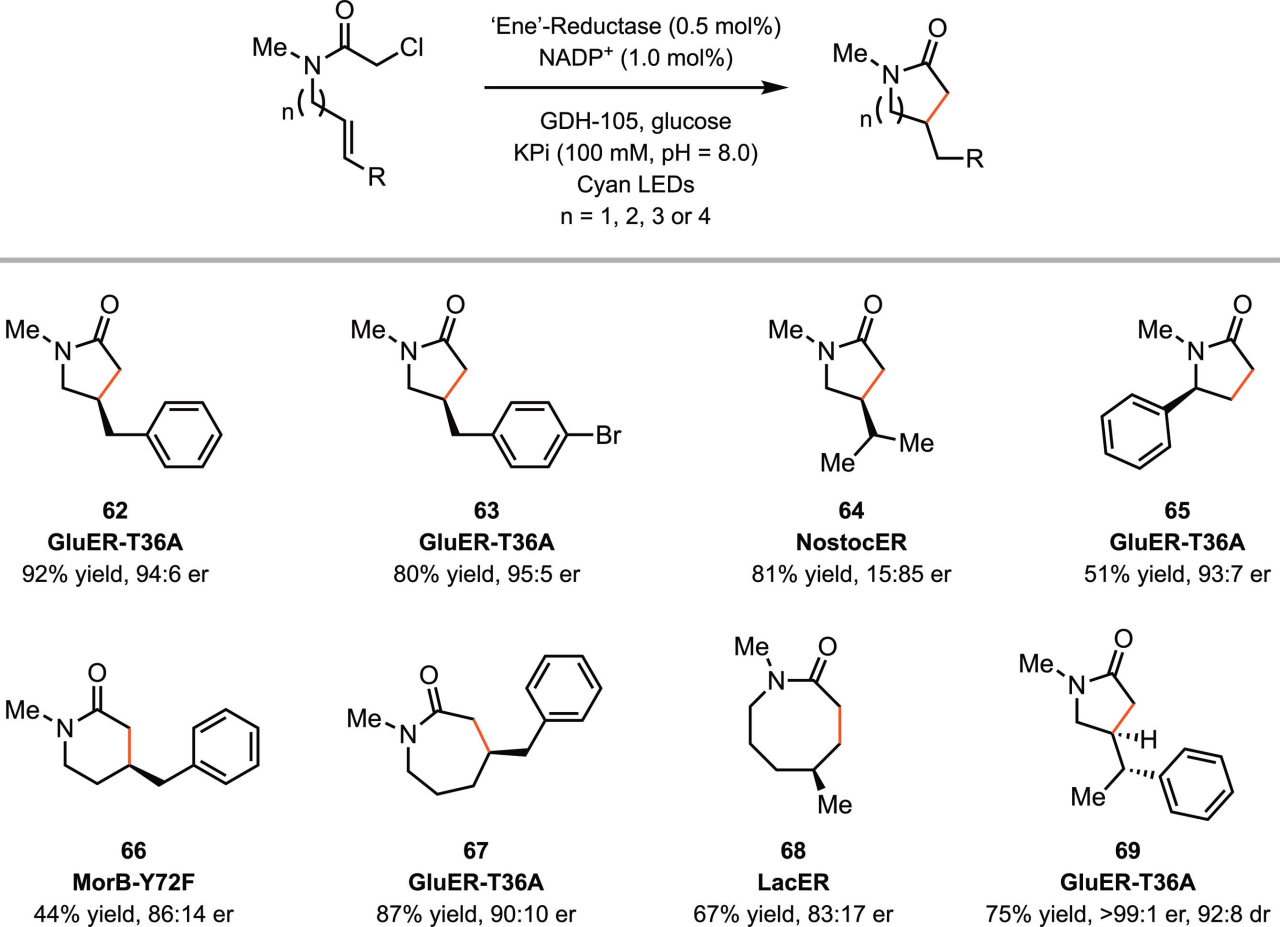
Representative substrate scope of the biocatalytic radical cyclization of α-chloroamides.

As a model for this reactivity, Hyster and coworkers targeted the development of a biocatalytic radical cyclization of α-chloroamides to afford β-stereogenic lactams (Biegasiewicz et al., 2019). The lactam motif is prevalent in medicinally valuable molecules (Vitaku et al., 2014), and the proposed synthesis would be distinct from existing biocatalytic approaches for generating N-heterocycles (France et al., 2016). Although this cyclization is well known in the radical literature, it is plagued by the preferential formation of the hydrodehalogenated and oligomerized product, and there are no known catalytic asymmetric variants (Curran & Tamine, 1991; Hiroi & Ishii, 2000). They realized that flavin hydroquinone (FMN_hq_) is a modest single-electron reductant [*E*_1/2_ = −0.45 V versus saturated calomel electrode (SCE)], making electron transfer to α-chloroamides (*E*_p/2_^red^ = −1.65 V versus SCE) thermodynamically challenging. The excited state of the flavin hydroquinone (FMN_hq_*) (*E*_1/2_ = −2.26 V versus SCE), however, should be capable of accomplishing this initial electron transfer (Scheme 13) (Ghisla et al., 1974; Massey et al., 1978; Warren et al., 2012). After investigation, they found that the cyclization occurs effectively when *Gluconobacter oxydans* ene-reductase (GluER) was used as the catalyst, and the reaction was irradiated with cyan light (497 nm) (Scheme 14). A variety of five-, six-, seven-, and eight-membered lactams with different substituent patterns were readily accessed. UV–vis and transient absorption spectroscopy established that radical formation occurs via excitation of an electron donor-acceptor complex that forms exclusively within the enzyme active site. This enzyme templated complex has a broad absorption band at λ = 500 nm, accounting for the reaction's wavelength preference. This represents a novel biocatalytic electron transfer mechanism that is distinct from the initially envisioned mechanism.

**Scheme 15.**
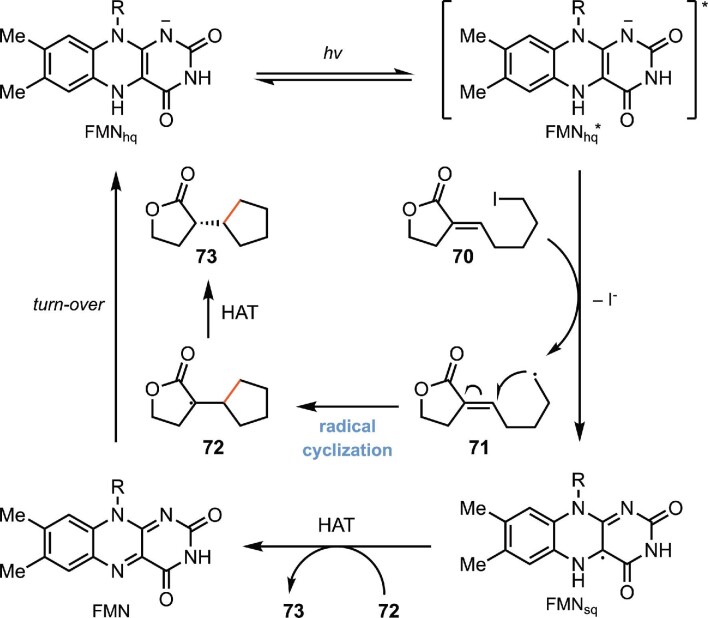
Proposed mechanism of the biocatalytic radical cyclization of alkyl iodides by photoexcited flavin proteins.

**Scheme 16.**
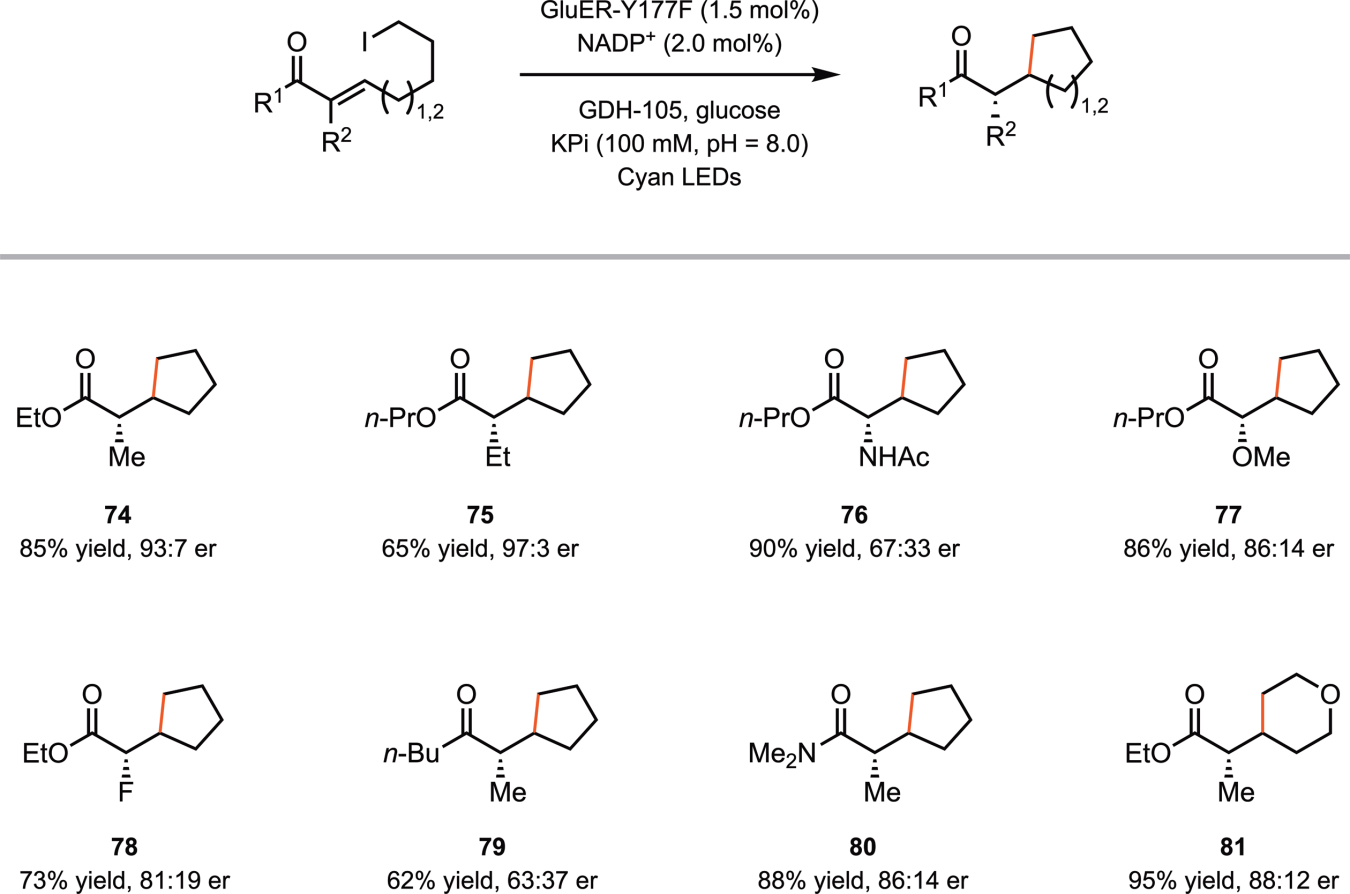
Representative substrate scope of the biocatalytic radical cyclization of alkyl iodides.

In addition to α-chloroamides, this strategy could also be applied to unactivated alkyl iodides (Clayman & Hyster, 2020). In contrast to α-halocarbonyl compounds, which possess comparably low reduction potentials and produce electrophilic radicals, unactivated alkyl iodides are more challenging to reduce and generate nucleophilic radicals. Similar to the case with α-chloroamides, Hyster and coworker found that upon binding to the protein active site, the substrate forms a charge-transfer complex (CT complex) with the fully reduced FMN_hq_. Photoexcitation of this CT complex facilitates the electron transfer between the alkyl iodide substrate and FMN_hq_, generating the primary alkyl radical, which involves the following radical cyclization process (Scheme 15). A variety of esters, amides, and ketones with an α-chiral center are efficiently synthesized. The reaction accommodates different substituents at the α-position, including alkyl substituents, an acetamide, an alkoxyl group, a fluorine atom, etc. In addition to 5-exo-trig cyclization to form a five-membered ring, 6-exo-trig cyclization could also be realized to provide a tetrahydropyran ring (Scheme 16).

### ERED-Catalyzed Redox-Neutral Radical Cyclization Reaction

**Scheme 17.**
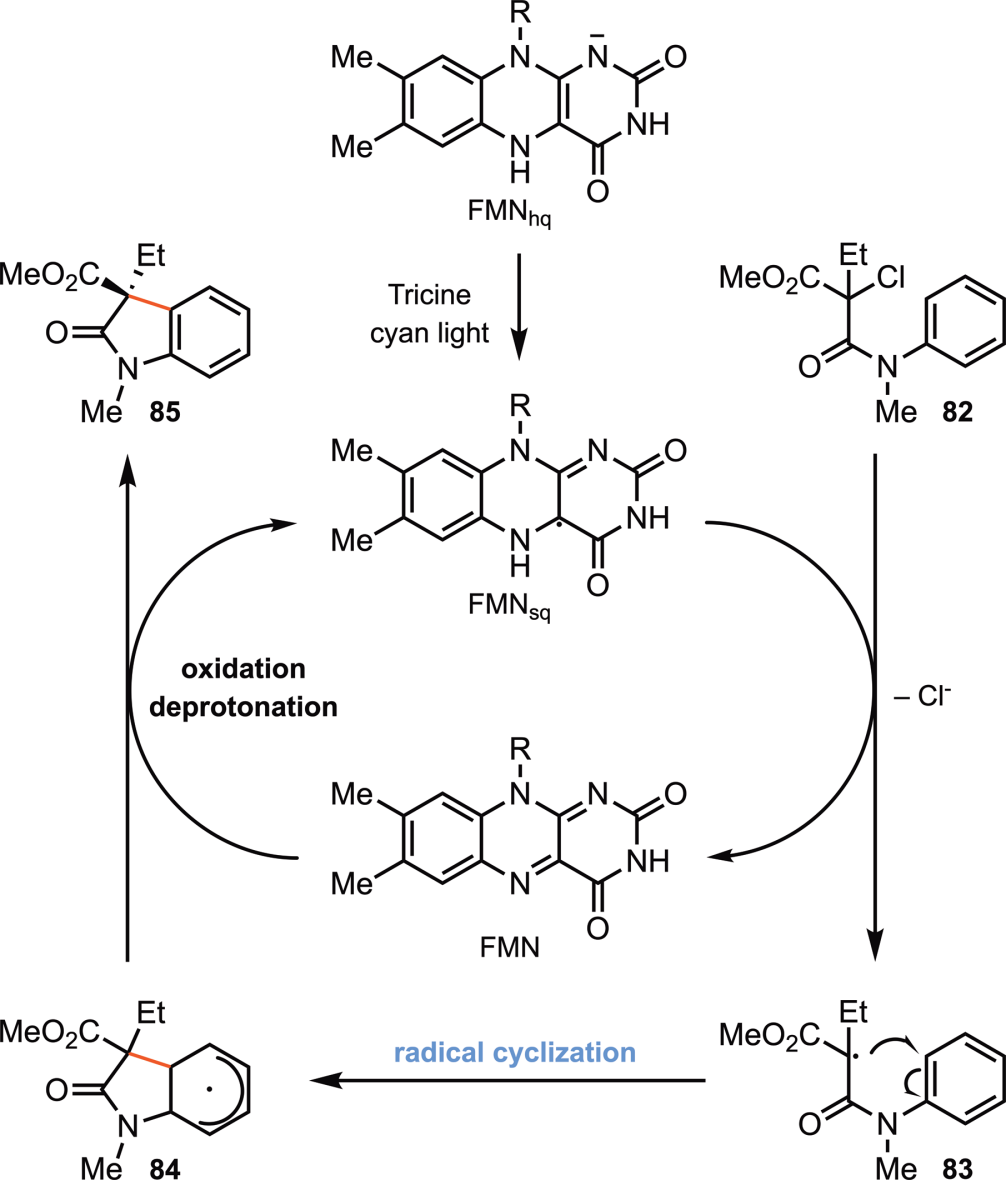
Proposed mechanism of the biocatalytic radical cyclization of α-halo-β-amides for the preparation of 3,3-disubstituted oxindoles catalyzed by photoexcited flavin proteins.

**Scheme 18.**
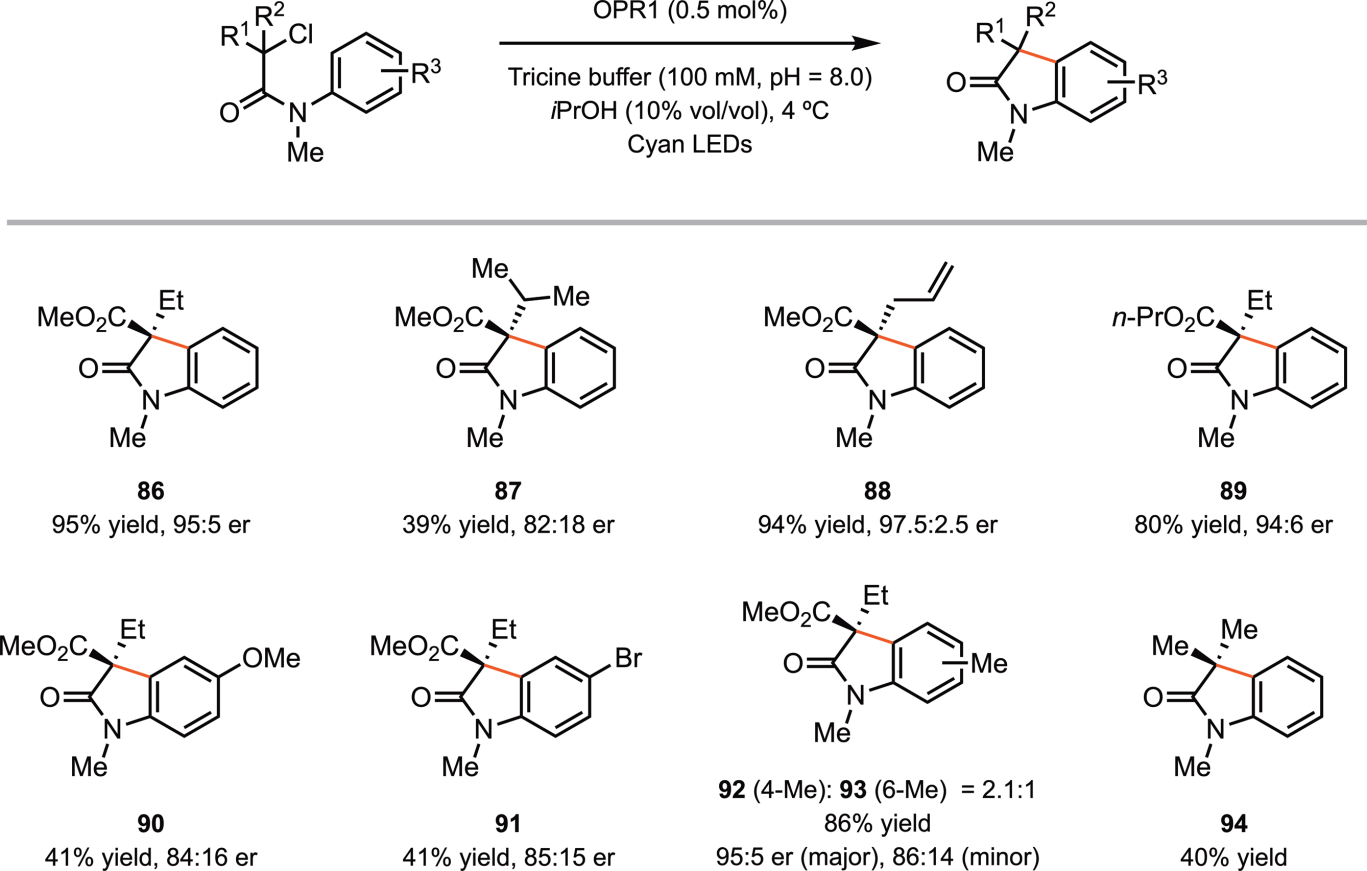
Representative substrate scope of the biocatalytic radical cyclization of α-halo-β-amides.

Hyster and coworkers developed a redox-neutral radical cyclization process to prepare 3,3-disubstituted oxindoles from the transformations discussed before, which are reductive radical cyclization reaction α-halo-β-amides (Black et al., 2019). 3,3-Disubstituted oxindoles are prevalent in medicinally valuable molecules, and there are no known methods for rendering this radical cyclization asymmetric (Ju et al., 2012; Zhou et al., 2010). The reaction was catalyzed by a EREDs (12-oxophytodienoate reductase, OPR1) and facilitated by cyan light. The proposed mechanism is shown in Scheme 17. Light and tricine buffer reduces FMN to FMN_sq_^–^, which reduces the substrate **82** to generate the radical intermediate **83** and FMN. Cyclization of **83** forms a reducing vinylogous amido radical intermediate **84** that can be oxidized by FMN to form the product **85** and regenerate FMN_sq_^–^. While FMN_sq_^–^ will undergo comproportionation under the reaction conditions, visible light irradiation provides a mechanism for rescuing flavin from catalytically inactive oxidation states to FMN_sq_^–^ thus representing a unique mechanism in photoenzymatic catalysis. This reaction tolerates a variety of substituents at the α-position of the amide (Scheme 18). Several ester substituents are accepted as well. Substrates with electron-donating and electron-withdrawing groups on the aromatic ring undergo the transformation successfully. When the electron-withdrawing ester group is removed from the substrate, the desired oxindole product is still observed.

## Conclusion

Insights as to how nature facilitates radical chemistry on structurally complex molecules in a highly selective manner highlight the prospect for enzymes to address fundamental challenges in the synthetic literature. These opportunities lie in the development of new enzyme enabled syntheses, where the synthesis of complex molecules and their analogs can be streamlined through the inclusion of enzymatic steps. Alternatively, by identifying the general strategies that nature uses to form and harness radical intermediates, new small molecule or enzymatic catalysts can be developed, which take inspiration from their analogs in nature. The fingerprints of enzymatic inspiration can be seen in the development of new photoenzymatic systems for radical reactions. Alternatively, the advent of small molecule hydrogen-bonding catalysts, which bind to radicals in similar strategies to enzymes, highlights how developments in one area of synthesis can spur innovations in another. We are optimistic that further discoveries in the biocatalytic radical cyclization arena will have broad implications in chemical synthesis.

## Funding

This work was financially supported by the NIGMS (R01 GM127703).

## Conflict of Interest

The authors declare no conflict of interest.
